# Theoretical study of interaction between temozolomide anticancer drug and hydroxyethyl carboxymethyl cellulose nanocarriers for targeted drug delivery by DFT quantum mechanical calculation

**DOI:** 10.1186/s13065-023-01029-7

**Published:** 2023-09-14

**Authors:** Masoumeh Shahi, Fatemeh Azarakhshi

**Affiliations:** 1 Department of Organic Chemistry, Faculty of Pharmaceutical Chemistry, Tehran Medical Sciences, Islamic Azad University, Tehran, Iran; 2 Department of Chemistry, Varamin-Pishva Branch, Islamic Azad University, Varamin, Iran

**Keywords:** Temozolomide, DFT, NBO analysis, HCM-cellulose

## Abstract

In this article for the first time the quantum calculations of 3-methyl-4-oxoimidazo[5,1-d][1,2,3,5]tetrazine-8-carboxamide (Temozolomide) in HCM-Cellulose Substrate are evaluated using the B3LYP/6-31G level of theory. The non-bonded interaction effects of the molecule Temozolomide, HCM-Cellulose on the electronic properties, chemical shift tensors and natural charge have also been detected. Natural bond orbital analysis (NBO) suggests that Temozolomide as an electron donor and HCM-Cellulose acted as an electron acceptor in the Temozolomide/HCM-Cellulose complex. The electronic spectra of the Temozolomide drug and Temozolomide/HCM-Cellulose complex in were calculated by Time-Dependent Density Functional Theory (TD-DFT) for the investigation of the adsorption effect of the Temozolomide drug over HCM-Cellulose on maximum wavelength. As a result, the feasibility of using HCM-Cellulose to deliver Temozolomide to diseased cells has been established.

**Supplementary Information:**

The online version contains supplementary material available at 10.1186/s13065-023-01029-7.

## Introduction

The current treatment for cancer includes chemotherapy, radiation and surgery. The purpose of chemotherapy and radiotherapy is to destroy cancerous cells. The effectiveness of a treatment is directly related to its ability to destroy cancer cells so that healthy cells in the body are not affected. The limiting factor for cancer chemotherapy is the non-selective use of cancer cell drugs [1, 2]. Also, during chemotherapy, some cells become resistant to the treatment, and to solve this problem, they either increase the dose of the drug during the treatment, or several drugs are used at the same time. However, with these measures, the drug's toxicity also increases. Different drug administration systems have been developed to reduce these side effects. Recently, many researches in the field of chemotherapy have been investigated for the use of drug carriers and nanodrug delivery systems. The purpose of the development of these systems is the controlled release of the drug, maintaining the concentration of the drug in the therapeutic range for a suitable period of time and the specific transfer of the drug to the target tissue. These nanomedicine systems include liposomes, micelles, nanoparticles, and polymeric nanocarriers [3, 4]. Nowadays, Due to the many advantages of nanostructures, such as the ability to carry several drugs at the same time and reducing toxicity with the aim of delivering drugs to cancer cells, these structures have attracted the attention of many researchers. These new drug delivery systems will improve the performance of the drug [5–7]. For many years, extensive research has been conducted in the field of using biodegradable polymers in drug delivery systems to target and control the rate of drug release. A biodegradable polymer drug release system can provide patient comfort and satisfaction by eliminating the side effects of drug use and reducing the number of times it is prescribed. Most of the drug delivery products based on biodegradable polymers are used in the form of injectable products, and the two categories of injectable drug delivery systems, particulate and formed in place, are more widely used. A drug delivery system based on a biodegradable polymer is designed in such a way that, in addition to obtaining a product with physical–mechanical properties appropriate to the tissue of the administration site in the body, the desired result of drug degradation and release is obtained [8–14]. Cellulose is a natural linear polymer with chloropyranose unit, which has biodegradable, non-toxic, recyclable, hydrophilic and safe properties. Cellulose is used in various industries such as wood and pharmaceutical and health industries. One of the wide applications of cellulose and its derivatives is in pharmaceuticals and cosmetics. Microcrystalline cellulose is the most widely used in pharmaceuticals. Cellulose ether and cellulose ester are two important derivatives of cellulose in the pharmaceutical industry, which can be referred to as hydroxypropyl methyl cellulose, hydroxypropyl cellulose, hydroxyethyl cellulose, carboxymethyl cellulose, sodium carboxy Methyl Cellulose. Cellulose and its derivatives in the formulation of drugs, in the preparation of tablet and capsule coatings, targeted drug carriers, encapsulation of drug particles, drug release control has very wide applications. The unique properties of these materials, including high tensile strength, high hardness, and photonic properties, make them attractive for use in many fields, especially in medicine and drug delivery. The construction of biological scaffolds used in tissue engineering, anticancer drugs and dental ceramics are among the applications of cellulose nanomaterials due to their high biocompatibility and rapid degradability [15–18].

Considering the complications caused by the use of drugs, especially anticancer drugs, it seems necessary to target drug delivery using nanoparticles. In recent years, a lot of attention has been paid to the preparation of nanoparticles as drug carriers, because nanoparticles can be used as a drug due to their control and slow release, particle size smaller than cells, biocompatibility. Extensive research and development is underway in the world to identify the applications of bio-nanocarriers in drug delivery, but theoretical studies on the ability of surface-modified cellulose to absorb drugs are limited [19, 20]. Therefore, the main goal of this research is to theoretically study the interaction of the anticancer drug Temozolomide on the surface of HCM-Cellulose as a drug delivery system using the DFT quantum mechanical calculation method. In 2018, Seyedah Mehsa Mousavi Langari, Maryam Nikzad and colleagues reviewed the types of nanocellulose and its applications in medicine. Nanocellulose is a unique natural substance extracted from lignocellulosic materials, which in recent years has attracted the attention of many researchers for medical applications due to its remarkable physical, chemical and biological properties such as biocompatibility, biodegradability and low toxicity. The purpose of the present study is to review the types of nanocellulose and their applications in medicine, including drug delivery, tissue engineering, implants and implantation of alternative substances in the body and antibacterial substances. In general, three types of nanocellulose, namely cellulose nanofibers, cellulose nanocrystals and bacterial cellulose, have been introduced, whose presence in medical products improves mechanical and biological properties and reduces toxicity. The purpose of the present study is to review the types of nanocellulose and their applications in medicine, including drug delivery, tissue engineering, implants and implantation of alternative substances in the body and antibacterial substances. In general, three types of nanocellulose, namely cellulose nanofibers, cellulose nanocrystals and bacterial cellulose, have been introduced, whose presence in medical products improves mechanical and biological properties and reduces toxicity. In 2015, the design and synthesis of nanocellulose functionalized with vitamin A was investigated and its ability to absorb aflatoxin B1. Firstly, cellulose nanoparticles were synthesized by acid hydrolysis and then conjugated to vitamin A with the help of a cross-linking device. In order to study the structure, the method of imaging with transmission electron microscope and Fourier transform infrared spectrometry was used. The amount of aflatoxin absorption was evaluated under different conditions of temperature, time, pH and concentration [25].

In 2021 Raluca Nicu and et al., studied on the functional materials based on nanocellulose for pharmaceutical properties. Researchers have special attention Nanocelluloses (NCs), with their characteristics, biocompatibility and their physicochemical properties [26]. In 2016, Mirdehghan and et al., investigated the interaction between conjugated nanocellulose with Aflatoxin drug. According to results, the decrease of pH led to increase of adsorption drug [27].

The present study aims to determine how Temozolomide absorbs HCM-Cellulose using DFT calculations at the B3LYP/6-31G* level of theory. Our work also included the study of, NBO analysis, electronic properties, NMR analysis and absorption spectrum of the compound Temozolomide, HCM-Cellulose and Temozolomide/HCM-Cellulose complex.

## Theoretical calculations

The calculation of quantum theoretical properties of Temozolomide, HCM-Cellulose, and Temozolomide/HCM-Cellulose complex was performed using Gaussian 09W program package [28] using the DFT method. The title compounds were first optimized, then frequency calculations were performed in gas phase to examine thermodynamic functions such as relative energies (E), free Gibbs energies (G), entropies (S), and enthalpies (H) [17, 18]. The NBO 5.G program was then used to calculate NBOs (natural bond orbitals) at the same level as a measure of inter-molecular delocalization or hyper-conjugation, which is a measure of second-order interactions. Temozolomide, HCM-Cellulose, and the Temozolomide/HCM-Cellulose complex were analyzed through NBO analysis to determine electronic properties like EHOMO, ELUMO, HOMO-LUMO energy gap (∆E gap), natural charges, molecular properties, dipole moment (μ), charge density, density of state (DOS). GaussView 09 software was used to visualize the optimized molecular structure, HOMO and LUMO surfaces [29]. In addition, NMR parameters such as chemical shift anisotropic (CSA) and chemical shift isotropic (CSI) for title structures based on B3LYP/6-31G* level experiments were calculated [21]. Temozolomide adsorption energy on HCM-Cellulose was computed according to the following formula:

$$ {\text{E}}_{{{\text{ad}}}} = {\text{E }}_{{\text{Temozolomide/HCM - Cellulose}}} - ({\text{E }}_{{{\text{Temozolomide}}}} + {\text{E }}_{{\text{HCM - Cellulose}}} ) $$

The energy of (E _Temozolomide/HCM-Cellulose_) represents the total energy of the Temozolomide/HCM-Cellulose complex, including both the compound Temozolomide and the compound HCM-Cellulose. The energy of (E _Temozolomide_) and (E _Temozolomide /HCM-Cellulose_) represents the total energy of their respective compounds.

## Results and discussion

### Optimized geometry

**Fig. 1:**
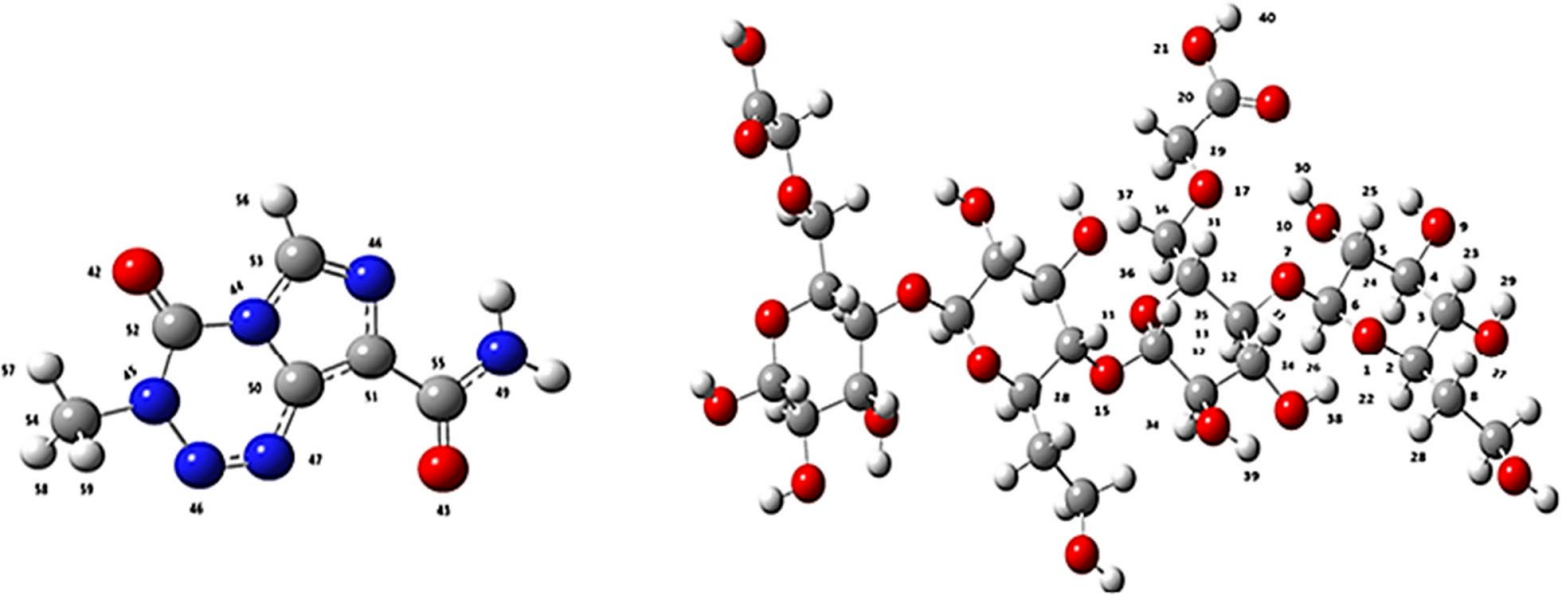
The optimized structures of the compounds Temozolomide and HCM-Cellulose using B3LYP/6-31G* level of theory

**Fig. 2:**
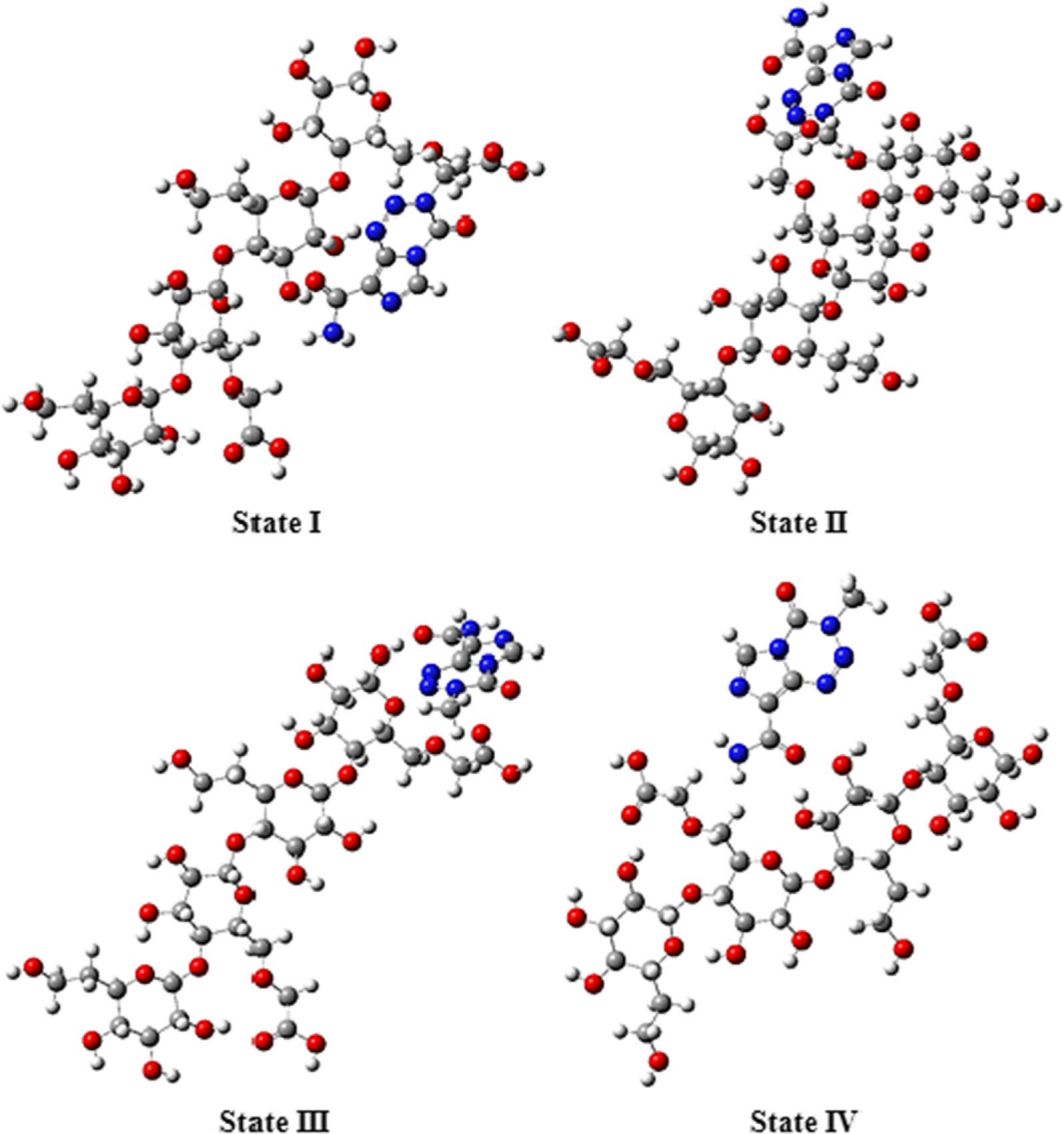
The four interaction of the Temozolomide with HCM-Cellulose optimized by HF/STO-3G

The optimized structures and frequency calculation of the Temozolomide, HCM-Cellulose and Temozolomide/HCM-Cellulose complex and the interactions of the Temozolomide with the HCM-Cellulose were performed by the DFT method at B3LYP/6-31G* level of theory. In Fig. 1 the optimized structures of the compounds Temozolomide and the HCM-Cellulose are shown. We have considered four interactions between the Temozolomide with the HCM-Cellulose. The four optimized states (I–V) were calculated at the HF/STO-3G basic set (Fig. 2).

The calculated values of electronic energy (E) for the four states I-IV were calculated: − 2,318,950.95, − 2,318,959.385, − 2,318,957.524, − 2,318,954.605 kcal/mol, respectively. The state II has the lowest energy value, as indicated by Table 1. Table 1 shows the differences in the DFT-calculated energies of molecules for a number of calculated I- IV conformations. The II conformation turns to be the lowest by energy, and its energy were chosen as the reference energy. Relative DFT energies ΔE for a number of stable conformations of HCM-Cellulose optimized by HF/STO-3G” was calculated. As can be seen from the data of Table 1, the II conformation should exceed significantly the contents of the molecules in other conformations.

**Table 1:** Shows the differences in the DFT-calculated energies (Relative energies) of molecules for a number of calculated I-IV conformations and the energy parameters for four interaction between the Temozolomide and HCM-Cellulose were optimized by HF/STO-3G method

Parameter	State I	State II	State III	State IV
ΔE (kcal/mol) Conformation	8.429	0.00	1.861	4.780
E_HOMO_ (eV)	− 8.606896	− 8.748608	− 8.596016	− 8.584864
E_LUMO_ (eV)	4.482832	4.021521	4.406944	3.987792
E g (eV)	13.089	12.770	13.002	12.572

In addition, Table 2 presents the thermodynamic parameters optimized by the HF/STO-3G method for the four interactions between Temozolomide and HCM-Cellulose. These parameters include the thermal energies (T), the thermal enthalpies (H), and the thermal free energies (G). The negative electron energy, enthalpies, and Gibbs energies of I-IV make them stable vibrational states. Based on the results, state II has a lower energy value than all the other states, making it more stable. Following this, we considered the interaction between Temozolomide and HCM-Cellulose at the state II and optimized using the B3LYP/6-31G* level of theory. Table 3 shows the thermochemical parameters for interaction of the compound Temozolomide with the HCM-Cellulose (state II) optimized using B3LYP/6-31G* method. A non-bonded interaction between Temozolomide and HCM-Cellulose results in lower Enthalpy and Gibbs energies. The energy values indicate that Temozolomide is less reactive and more stable with the presence of HCM-Cellulose.

**Table 2:** The thermodynamic parameters for four interaction of the Temozolomide/HCM-Cellulose complex at HF/STO-3G level of theory calculated in kcal/mol

State	G (kcal/mol)	H (kcal/mol)	T (kcal/mol)	S (kcal/mol)
I	− 2,318,297.33	− 2,318,177.731	− 2,318,178.323	0.4011
II	− 2,318,304.235	− 2,318,185.485	− 2,318,186.077	0.3983
III	− 2,318,301.629	− 2,318,183.762	− 2,318,184.355	0.3953
IV	− 2,318,298.064	− 2,318,181.001	− 2,318,181.594	0.3926

**Table 3:** The thermdynamic parameters of Temozolomide, HCM-Cellulose and Temozolomide/HCM-Cellulose complex (II) at B3LYP/6-31G* level of theory calculated in kcal/mol

Parameter	Temozolomide	HCM-Cellulose	Temozolomide/HCM-Cellulose(II)
G **(**kcal/mol**)**	− 446,206.549	− 1,915,763.612	− 2,361,969.514
H **(**kcal/mol**)**	− 446,175.1026	− 1,915,662.842	− 2,361,850.851
T **(**kcal/mol**)**	− 446,175.6956	− 1,915,663.434	− 2,361,851.443
S **(**kcal/mol**)**	0.1055	0.3380	0.3981

### Electronic properties and frontier analysis

Analysis of the frontier molecular orbitals (FMO) plays a major role in understanding electronic properties [22, 30]. This study examined the electronic properties of Temozolomide when it interacts with HCM-Cellulose using B3LYP at the level of 6-31G* (Table 4 the results of the calculations). According to Table 4, the chemical reaction between Temozolomide and HCM-Cellulose has an exothermic nature due to its negative adsorption energy (E_ad_ = − 0.743 eV).

**Table 4:** The calculated electronic properties of Temozolomide, HCM-Cellulose and Temozolomide/HCM-Cellulose complex at B3LYP/6-31G* level of theory calculated

Property	Temozolomide	HCM-Cellulose	Temozolomide/HCM-Cellulose
Dipole moment (Debye)	3.3419	7.7945	6.8985
$E (\mathrm{kcal}/\mathrm{mol})$	− 446,271.178	− 1,916,238.079	− 2,362,526.404
E_HOMO_ (eV)	− 6.862288	− 6.628368	− 6.624288
E_LUMO_ (eV)	− 2.454256	− 0.501568	− 2.579921
E_g_ (eV)	4.408032	6.12681	4.044368
E_ad_ (eV)	–	–	− 0.74324
E_I_ (eV)	6.862288	6.628368	6.624288
E_A_ (eV)	2.454256	0.501568	2.579921
$\chi$(eV)	4.658272	3.564968	4.602104
$\eta$(eV)	2.204016	3.063411	2.022184
$\mu$(eV)	− 4.658272	− 3.564968	− 4.602104
ω (eV)	4.922717	2.074328	5.236754
S (eV^−1^)	6.170554	4.439511	6.725401

**Fig. 3:**
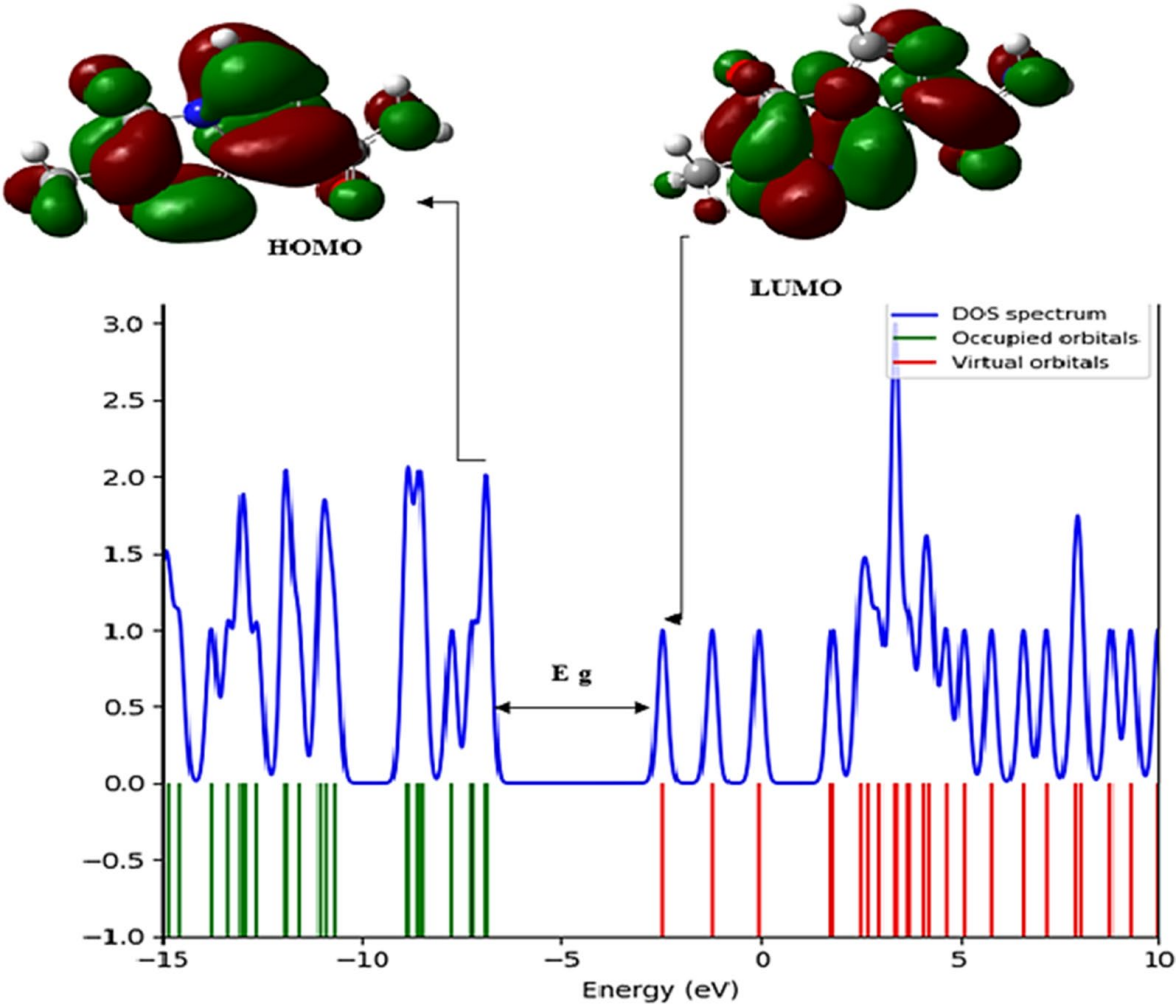
Temozolomide HOMO and LUMO orbitals calculated and DOS plotted

**Fig. 4:**
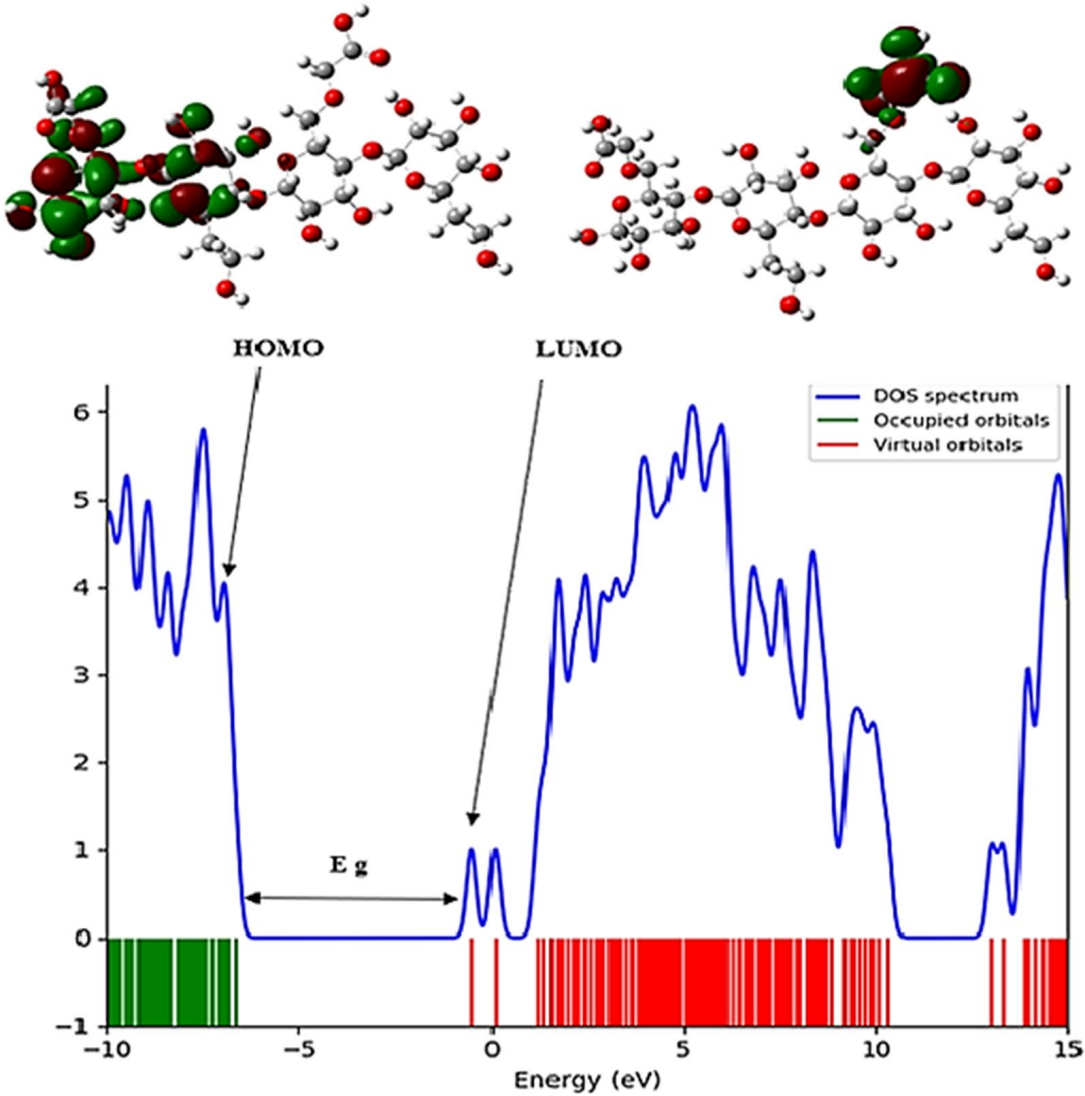
HCM-Cellulose HOMO and LUMO orbitals calculated and DOS plotted

The FMO analysis of Temozolomide shows that the HOMO orbital spreads over the entire molecule, while the LUMO orbital focuses on the C=C, C=N, C=O, N=N, and NH groups, and also spreads over the entire molecule (Fig. 3). Hence, in Temozolomide, pi (p) bonds and nitrogen lone pairs are involved in transferring charge between the HOMO and LUMO. It can be seen in Fig. 4 that the LUMO orbitals focus on the carboxylic acid group while the HOMO orbitals focus on the HCM-Cellulose half structure. Upon adsorption of Temozolomide onto HCM-Cellulose, the LUMO/HOMO energy gap is 4.044 eV, which decrease from the original Temozolomide Eg value of 4.408 eV.

**Fig. 5:**
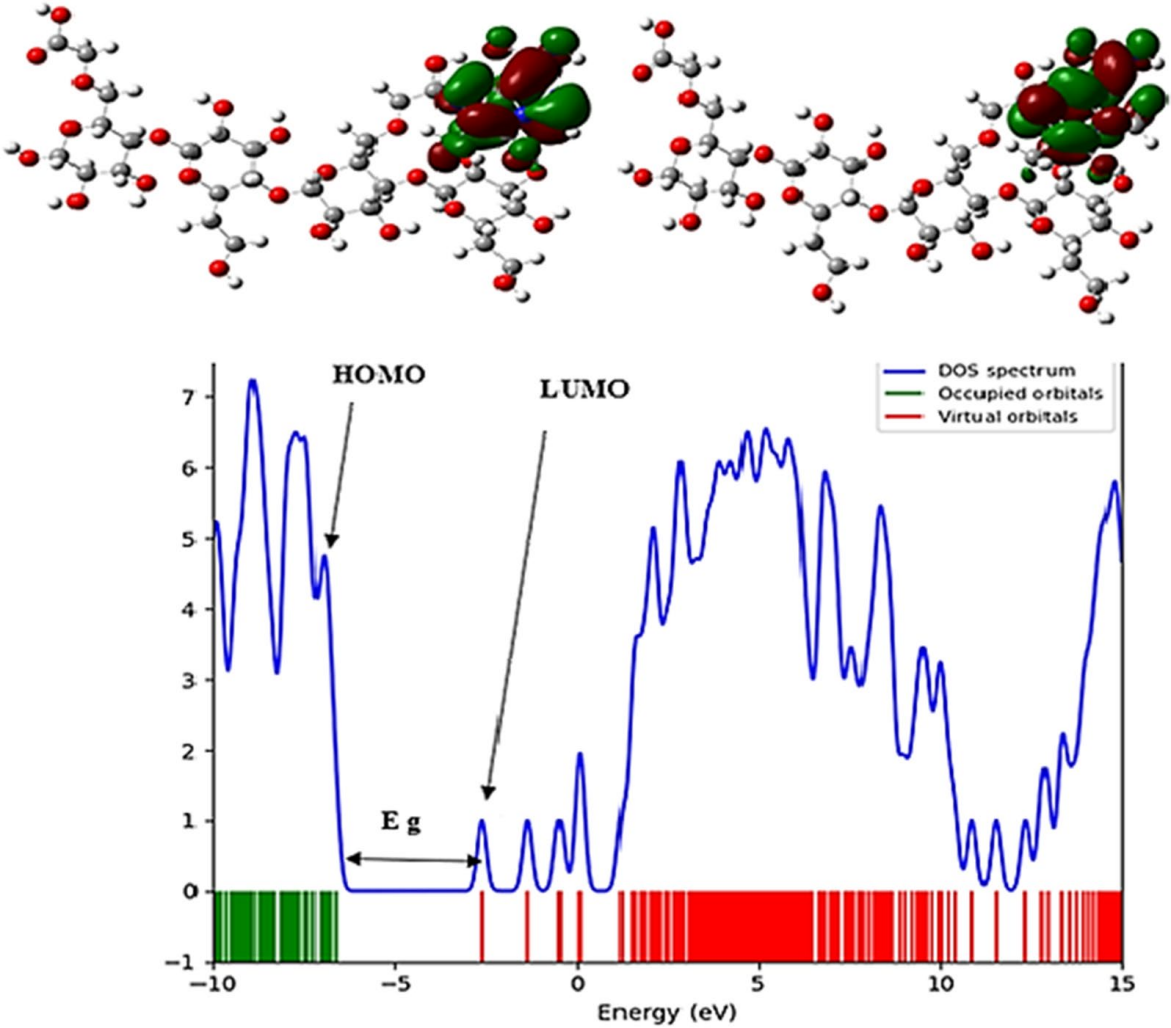
Temozolomide/HCM-Cellulose HOMO and LUMO orbitals calculated and DOS plotted

Additionally, DOS plots depict changes in energy gaps. As seen in (Fig. 5) of the FMO analysis of the Temozolomide/HCM-Cellulose complex, the HOMO and LUMO orbitals mostly focus on the Temozolomide form as a whole. The (Table 4) provides a detailed description of ionization potential (I), electron affinity (A), global hardness (η), electronegativity (χ), electronic chemical potential (µ), electrophilicity (ω) and chemical softness (S) of Temozolomide, HCM-Cellulose and Temozolomide/HCM-Cellulose complex. Ionization potential (I) is directly related to HOMO's energy, while electron affinity (A) is directly related to LUMO's energy [23, 24].

Based on the energy gap between HOMO and LUMO, the global hardness (η) can be determined. Molecules with a small energy gap are highly chemically reactive, have low kinetic stability, and are soft, while molecules with a large energy gap are hard. Based on global hardness values (η), Temozolomide, HCM-Cellulose and Temozolomide/HCM-Cellulose have 2.204 eV, 3.063 eV, and 2.022 eV respectively.

DFT method is a useful method to check the characteristics of chemical structures based on the reactivity index of molecules. The results of B3LYP/6-31G* calculations show that when the drug molecule is placed in the presence of the polymer, the energy gap value in the drug-polymer complex (Eg = 4.044 eV) compared to the energy gap in the temozolamide drug molecule alone (Eg = 4.408 eV) has decreased. On the other hand, the reactivity of a molecule is related to its energy gap [23, 24]. Examining the energy gap of molecular orbitals (ELUMO-EHOMO) shows that a soft molecule has a small energy gap and a hard molecule has a large energy gap. Stabilizing orbital interactions increase by decreasing the energy level of the electron acceptor orbital and increasing the energy level of the electron donor orbital. In addition, electron delocalization is confirmed by changing the population of electron donor and acceptor orbitals. Also, in the functionalized temozolamide-nanocellulose drug complex, compared to the drug alone, the hardness parameter, electronegativity and ionization energy have been reduced by reducing the Eg energy gap. The results of the calculations show that the energy gap in the drug-nanocarrier mixture is slightly reduced compared to the drug alone. This can be explained by the polarity and hydrophilicity of the drug-nanocarrier complex compared to the drug alone.

The dipole moments of Temozolomide, HCM-Cellulose and Temozolomide/HCM-Cellulose are 3.3419, 7.7945, 6.8985 Debye, respectively. Adsorption of Temozolomide onto HCM-Cellulose increases the dipole moment of Temozolomide to 6.8985 Debye. In molecular systems, atomic charges have a significant effect on the polarizability, dipole moment, electronic structure, and related properties. According to the B3LYP/6-31G* level of theory, the NBO charges have been calculated for equilibrium geometry of Temozolomide, HCM-Cellulose and Temozolomide/HCM-Cellulose. In Table 5, (atoms are labelled in Fig. 1), the calculated natural charges for selected atoms are shown. The natural charges of all HCM-Cellulose atoms changed significantly after interaction with Temozolomide. Based on the results, the values of charges for the H22-H31 and H37-H39 atoms of the HCM-Cellulose are 0.132e, 0.129e, 0.123e, 0.144e, 0.120e, 0.162e, 0.156e, 0.411e, 0.445e, 0.149e, 0.138e, 0.444e and 0.411e respectively, whereas in the Temozolomide/HCM-Cellulose complex, charges values are − 0.435e, − 0.509e, − 0.238e, − 0.225e, − 0.240e, − 0.234e, − 0.233e, − 0.482e, − 0.563e, − 0.562e, 0.540e, 0.193e and 0.843e. In HCM-Cellulose, the natural charge of the atoms C5, C19, C20 and O21 are approximately 0.228e, 0.260e, 0.571e and − 0.134e respectively, while in the Temozolomide/HCM-Cellulose complex, the charge values are 0.207e, 0.234e, 0.594e and − 0.112e. After non-bonded interactions with HCM-Cellulose, Temozolomide atoms also display changes in natural charges (Table 5). Temozolomide atoms also exhibit a change in natural charges following non-bonding HCM-Cellulose interactions (Table 5). Due to the interaction with HCM cellulose, all Temozolomide atoms had their charges significantly changed. The charge values for Temozolomide atoms O43, N45-49, C50, C53, C54, C55, H57 and H58 are − 0.503e, 0.376e, 0.316e, − 0.523e, 0.013e, − 0.760e, 0.492e, 0.237e, − 0.312e, 0.576e, 0.210e, and 0.193e respectively, after interaction with HCM-Cellulose, the charge values are − 0.563e, − 0.358e, 0.347e, − 0.533e, 0.003e, − 0.018e, 0.540e, 0.467e, 0.285e, 0.605e, 0.193e, and 0.211e, respectively. Among all atoms, the natural charge of the N atoms of the amide group in Temozolomide changed considerably after interaction with HCM-Cellulose. Therefore, the change in atomic charges from the interaction of HCM-Cellulose with Temozolomide induces a dipole moment for HCM-Cellulose in the Temozolomide/HCM-Cellulose complex. In other words, it illustrates the charge transfer and non-bonded interaction between Temozolomide and HCM-Cellulose.

**Table 5:** Calculated NBO charges and NMR parameters (ppm) including CS^I^ and CS^A^ for the selected atoms in Temozolomide, HCM-Cellulose, and Temozolomide/HCM-Cellulose

Atoms	HCM-Cellulose			Temozolomide			Temozolomide/HCM-Cellulose		
	Charge	CS^I^	CS^A^	Charge	CS^I^	CS^A^	Charge	CS^I^	CS^A^
1 O	− 0.546704	225.3934	65.0195	–	–	–	− 0.55053	225.5325	69.3592
2 C	0.251221	116.2507	53.4429	–	–	–	0.249805	116.2029	53.6695
3 C	0.277841	115.6254	28.5322	–	–	–	0.280875	117.2745	30.9183
4 C	0.221811	114.4354	20.263	–	–	–	0.224365	114.0017	18.7956
5 C	0.228732	117.6225	23.8495	–	–	–	0.207749	117.4201	16.8274
6 C	0.495289	84.6236	22.8412	–	–	–	0.502879	85.4078	22.2159
7 O	− 0.508161	238.3235	55.3899	–	–	–	− 0.509561	238.4410	61.3504
8 C	0.026525	150.7704	34.4676	–	–	–	0.025877	150.2514	35.4586
9 O	− 0.25156	297.3756	44.7762	–	–	–	− 0.257258	295.7038	48.6515
10 O	− 0.238376	300.5355	38.7607	–	–	–	− 0.234099	299.4512	36.2683
11 O	− 0.518134	247.8496	51.513	–	–	–	− 0.515772	248.2338	50.5650
12 C	0.259269	118.7769	55.4901	–	–	–	0.249511	118.5745	55.4077
13 C	0.254529	102.0348	42.2622	–	–	–	0.263474	103.4642	45.3202
14 C	0.20730	113.9545	13.1123	–	–	–	0.197978	113.1510	13.0813
15 O	0.517705	240.8107	50.3009	–	–	–	0.517732	240.1732	49.9372
16 C	0.2526	115.0507	65.6252	–	–	–	0.252914	117.0804	66.6162
17 O	− 0.465952	302.4619	43.3318	–	–	–	− 0.468779	304.383	47.8446
18 C	0.248306	61.1363	58.6559	–	–	–	0.250502	116.8695	58.7738
19 C	0.26022	123.0762	71.9808				0.234599	121.6419	63.0856
20 C	0.571372	27.5468	91.554				0.594905	24.6363	100.4042
21 O	− 0.134916	142.7448	176.5933				− 0.112916	121.9330	136.2371
22 H	0.13213	28.6933	4.0394				− 0.435311	28.8003	4.4054
23 H	0.12961	28.6498	2.9545				− 0.509378	28.3994	5.9867
24 H	0.123419	28.8892	4.1677				− 0.238703	29.0847	4.6628
25 H	0.144579	28.9152	3.4707				0.225848	30.1570	4.4939
26 H	0.120356	27.9634	4.2006				− 0.240728	27.9823	3.3749
27 H	0.162731	30.2756	5.3225				0.234068	30.3168	4.5427
28 H	0.156352	30.1602	3.7440				− 0.233241	29.9782	2.4853
29 H	0.411424	30.0362	16.2983				− 0.482377	30.6185	13.9885
30 H	0.445441	28.1936	25.3884				− 0.563044	27.5125	24.3871
31 H	0.149679	28.4230	4.2292				− 0.562966	28.5843	5.2403
32 H	0.134011	29.0225	4.9448				− 0.358727	28.9969	6.0565
33 H	0.130793	28.7801	5.1315				− 0.533054	28.8713	5.0172
34 H	0.133049	28.9038	2.6578				− 0.347772	28.9583	2.8597
35 H	0.140288	27.4412	7.0319				− 0.003101	27.4710	7.2559
36 H	0.148233	28.6119	7.8663				− 0.018522	28.7485	8.2718
37 H	0.138884	28.5731	3.4752				0.540296	28.6248	4.8241
38 H	0.444196	27.8760	28.3410				0.193304	28.0902	27.6647
39 H	0.411519	30.1553	17.4950				0.843033	30.1964	17.2357
40 H	0.421182	26.4750	11.9286				0.473643	21.1140	20.0994
41 O	− 0.454563	− 47.9148	512.5939				− 0.509378	− 20.3329	489.7466
42 O				− 0.48595	42.2236	432.4995	− 0.482377	24.4632	463.7483
43 O				− 0.50332	− 0.5766	537.7157	− 0.563044	12.7927	488.4237
44 N				− 0.55066	74.7740	150.9721	− 0.562966	74.1989	149.8885
45 N				− 0.37681	45.4537	161.8028	− 0.358727	45.2543	168.070
46 N				− 0.52347	− 11.0177	383.1138	− 0.533054	− 11.1776	383.0547
47 N				− 0.31661	− 92.1203	477.4035	− 0.347772	− 96.4220	481.0913
48 N				− 0.01317	− 144.7955	399.2429	− 0.003101	− 149.9911	379.4819
49 N				− 0.76061	159.5952	95.7293	− 0.018522	158.9593	95.7450
50 C				0.49246	59.9834	94.2179	0.540296	58.9789	97.7446
51 C				0.19434	62.9905	93.6961	0.193304	64.2087	95.9536
52 C				0.83314	57.5312	67.4470	0.843033	58.2438	68.0464
53 C				0.23794	66.9839	96.5516	0.467776	67.7003	97.9498
54 C				− 0.31296	155.4537	46.5400	0.285242	152.0157	47.1859
55 C				0.57653	39.2511	99.7280	0.60530	37.1299	106.092
56 H				0.20610	24.1137	4.3447	0.212824	24.1491	3.9622
57 H				0.21062	27.8165	5.3867	0.193608	28.3405	7.0327
58 H				0.19307	28.4505	7.9489	0.211201	28.2929	8.4974
59 H				0.19309	28.4497	7.9479	0.199809	28.2096	5.6820

### NMR analysis

In this research, the parameters of chemical coverage (CS), in order to obtain structural information, dynamic behavior and intramolecular interactions for the optimal structures of the drug temozolamide and HCM-Cellulose and the drug-HCM-Cellulose complex have been calculated using the gauge-including atomic orbital (GIAO) method. Chemical shift isotropic and anisotropic shifts were calculated for selected atoms in the compound Temozolomide, HCM-Cellulose and Temozolomide/HCM-Cellulose complex using the B3LYP/6-31G* level of theory. The chemical shift tensors (ppm) are shown in (Table 5). Based on the results, the values of CSI for the C3, O9, C16, O17, C19, C20, O21, H25, H30, H40, and O41 atoms of the HCM-Cellulose are 115.62, 297.37, 115.05, 302.46, 123.07, 27.54, 142.74, 28.91, 28.19, 26.47, − 47.91 ppm respectively, whereas in the Temozolomide/HCM-Cellulose complex, CSI values are 117.27, 295.70, 117.08, 304.38, 121.64, 24.63, 121.93, 30.15, 27.51, 21.11, − 20.33 ppm. A maximum change in CSI is observed for the O41 atom rather than for other atoms in HCM-Cellulose. Additionally, the CSA of O1, C5, O7, O9, C13, O17, C19, O20, O21, H22, H29, H40, and O41 atoms are 65.01, 23.84, 55.38, 44.77, 42.26, 43.33, 71.98, 91.55, 176.59, 2.95, 16.29, 11.92, 512.59 ppm respectively, whereas in the Temozolomide/HCM-Cellulose complex they are 69.35, 16.82, 61.35, 48.65, 45.32, 47.84, 63.08, 100.40, 136.23, 5.98, 13.98, 20.09, 489.74 ppm respectively. The change in the values of CSI and CSA for other oxygen, nitrogen, carbon and hydrogen atoms of the HCM-Cellulose is also observed after non-bonded interaction with the molecule Temozolomide. For the carboxylic acid atoms of the HCM-Cellulose, when interacting with Temozolomide, the highest CSI and CSA values refer to O21 and O41. In the Temozolomide compound, the CSI values for O42, O43, N48, N48, C51, C54 and C55 atoms are respectively 42.22, − 0.57, − 92.12, − 144.79, 62.99, 155.43, 39.25 ppm, while in the Temozolomide/HCM-Cellulose complex, 24.46, 12.79, − 96.42, − 149.99, 64.20, 152.01, 37.12 ppm respectively. As compared to the other atoms in the Temozolomide structure, O42 and O43 have the highest changes in CSI due to being close to the HCM-Cellulose in the non-bonded interaction, and the value of CSA changes from 432.46 and 537.71 to 463.74 and 488.42 respectively. Additionally, the CSA of N45, N47, N48, C50, C51, C55, H56 and H59 atoms are 161.80, 477.40, 399.24, 94.20, 93.69, 99.73, 4.34, 7.94 ppm respectively, whereas in the Temozolomide/HCM-Cellulose complex they are 168.07, 481.09, 379.48, 97.74, 95.95, 106.09, 3.96, 5.68 ppm respectively. CSI and CSA values of the other atoms of Temozolomide also change after the interaction (Table 5).

### Molecular electrostatic potential (MEP) analysis

**Fig. 6:**
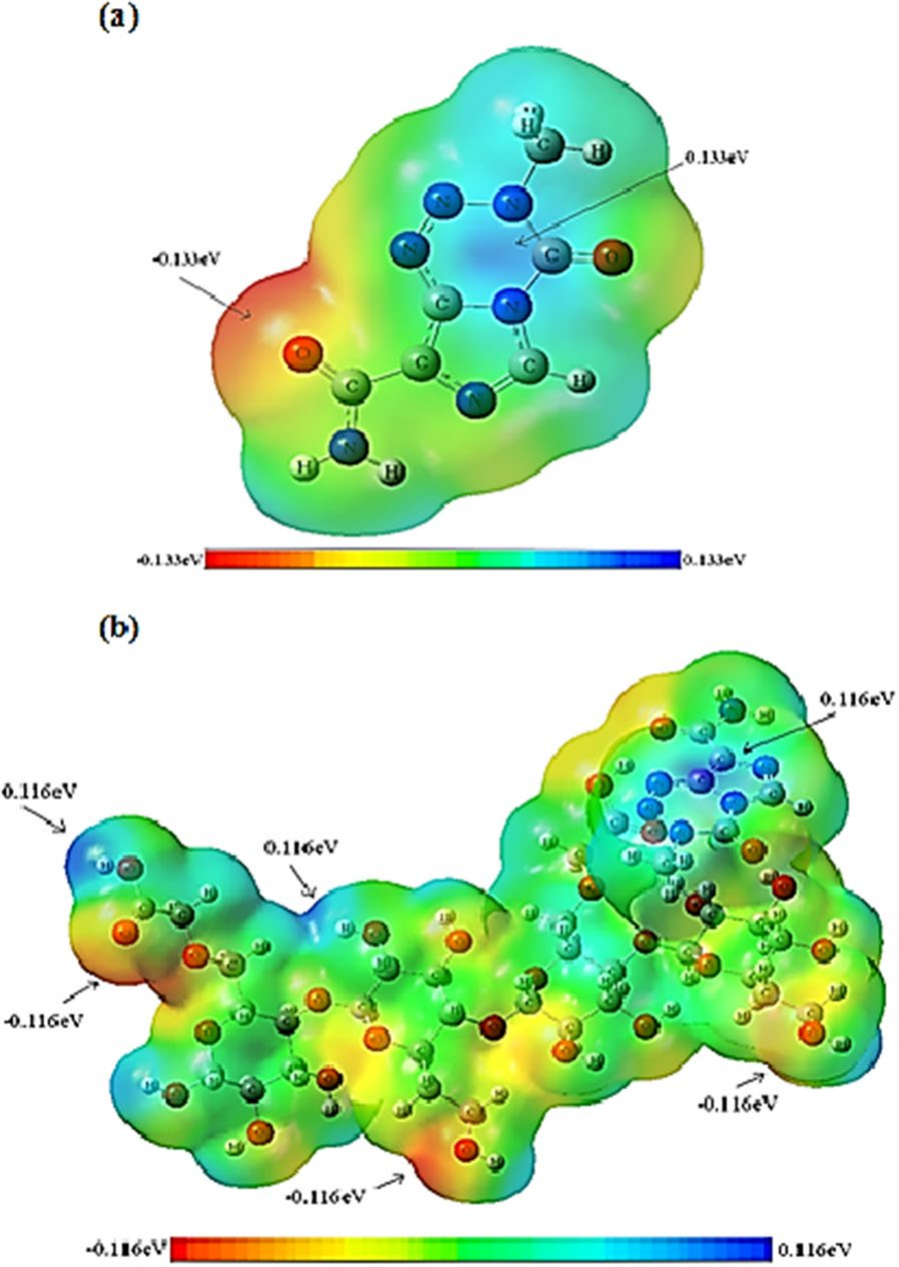
Generated molecular electrostatic potential (MEP) maps of temozolamide drug (**a**) and temozolamide-nanocellulose complex (**b**). The negative (red, orange, and yellow) regions are related to electrophilic reactivity, whereas positive (green and blue) regions accompany nucleophilic reactivity

MEPs (molecular electrostatic potentials) reveal the electronic density of molecules and are used for electrophilic and nucleophilic attack and reaction sites [23]. It was represented by different colors according to the electrostatic potential of the surfaces. Negative regions on MEP are colored red, indicating electrophilic reactivity, while positive regions are painted blue, indicating nucleophilic reactivity, and green, neutral reactivity. A theoretical calculation using the B3LYP/6-31G* level of energy was used to estimate the MEPs of the Temozolomide/HCM-Cellulose complex (Fig. 6) shows the molecular electrostatic potential (MEP) calculation for Temozolomide and Temozolomide/HCM-Cellulose. According to the MEP of Temozolomide, the positive charge density and the blue spectrum occur on the hydrogen atoms attached to Nitrogen and Nitrogens of tetrazine ring, while the negative charge density occupies the oxygen atom of the carboxamide groups. In the MEP of Temozolomide/HCM-Cellulose complex, the positive charge density occur on the hydrogen atoms associated with oxygen or nitrogen and tetrazine ring has a blue color, while the negative charge density occupies all the oxygen atoms. Green color indicates zero and neutral potential areas in Temozolomide/HCM-Cellulose and Temozolomide. The MEP (Molecular Electrostatic Potential) is plotted in Fig. 6 along with a colored bar indicating the maximum and minimum electrostatic potential presented.

### NBO analysis

Molecular systems can be studied by NBO analysis by examining intra- and intermolecular bonds and interactions between them. A conjugative electron transfer between donor and acceptor orbitals occurs when electrons delocalize from donor to acceptor orbitals. It is estimated that the stabilization energy involving the delocalization i → j will be as follows for each donor (i) and acceptor (j) [24]:

$$E\left(2\right)=\Delta Eij=qi \frac{F\left(i,j\right)2}{Sj-Si}$$

E(2) is a measure of how much electrons participate in the resonance between atoms in a molecule. When E(2) is high, electron donors are more likely to donate electrons to electron acceptors [33]. NBO analysis of Temozolomide/HCM-Cellulose was conducted at the B3LYP/6-31G* level of theory as summarized in Table 6. Based on the NBO analysis, the oxygen lone pairs (LPs O1 and O9) of the HCM-Cellulose overlap to the anti-bonding orbital σ*(C54-H58) and π* N46-C53 of the Temozolomide in the Temozolomide/HCM-Cellulose complex.

**Table 6:** The donor–acceptor interactions and second-order perturbation energies (E(2), kcal/mol) related to charge transfer between Temozolomide and HCM-Cellulose

Donor NBO (i)	Acceptor NBO (j)	E(2) kcal/mol	E(j)-E(i) a.u	F(i,j) a.u
*From HCM-Cellulose to Temozolomide*				
σ C5-H25	π* O42-C52	0.24	0.48	0.010
π C20-O41	π* N47-N48	0.15	0.32	0.007
σ O21-H40	σ* O43-C55	0.25	1.30	0.016
σ O21-H40	π* O43-C55	0.27	0.74	0.014
LP (1) O1	σ* C54-H58	0.14	0.98	0.011
LP (1) O9	π* N46-C53	0.11	0.58	0.008
LP (2) O9	π* N46-C53	0.20	0.28	0.007
*From Temozolomide to HCM-Cellulose*				
π O42-C52	σ* C3-H23	0.80	0.88	0.024
π O42-C52	σ* C5-H25	0.10	0.86	0.008
σ O43-C55	σ* O21-H40	0.50	1.45	0.024
π O43-C55	σ* O21-H40	2.66	0.79	0.041
π N49-C55	σ* O21-H40	0.20	1.26	0.015
LP(1) O42	σ* C3-H23	0.72	1.18	0.026
LP(1) O43	σ* O21-H40	12.26	1.07	0.103
LP(2) O43	σ* O21-H40	14.80	0.70	0.092
LP(1) N47	σ* O21-H40	0.27	0.81	0.013

As a result of this overlap, electron charge transfer takes place as σ C5-H25 → π* O42-C52, π C20-O41 → π* N47-N48, σ O21-H40 → σ* O43-C55, σ O21-H40 → π* O43-C55, LP(1) O1 → σ* C54-H58 and LP(1) O9 → π* N46-C53 and LP(2) O9 → π* N46-C53 with stabilization energies E(2) 0.24 kcal/mol, 0.15 kcal/mol, 0.25 kcal/mol, 0.27 kcal/mol, 0.14 kcal/mol, 0.11 kcal/mol, and 0.20 kcal/mol, respectively. According to NBO analysis, the main interaction is σ O21-H40 → σ* O43-C55 and σ O21-H40 → π* O43-C55 transition with resonance energy values (E(2)) of 0.27 kcal/mol and 0.25 kcal/mol, respectively. Therefore, HCM-Cellulose acts as an electron donor and Temozolomide drug acts as an electron acceptor.

NBO calculation indicates that electron charge transfer takes place as π O42-C52 → σ* C3-H23, π O42-C52 → σ* C5-H25, σ O43-C55 → σ* O21- H40, π O43-C55 → σ* O21- H40, π N49-C55 → σ* O21- H40, LP(1) O42 → σ* C3-H23, LP(1) O43 → σ* O21-H40, LP(2) O43 → σ* O21-H40 and LP(1) N47 → σ* O21-H40 with stabilization energies E(2) 0.80 kcal/mol, 0.10 kcal/mol, 0.50 kcal/mol, 2.66 kcal/mol, 0.20 kcal/mol, 0.72 kcal/mol, 12.26 kcal/mol, 14.80 kcal/mol, and 0.27 kcal/mol, respectively. The main interaction is LP(1) O43 → σ* O21-H40 and LP(2) O43 → σ* O21-H40 transition with resonance energy values (E(2)) of 12.26 kcal/mol and 14.80 kcal/mol, respectively. As results, Temozolomide drug acts as an electron donor and HCM-Cellulose acts as an electron acceptor.

### Structural parameters

Structural parameters calculated using B3LYP/6-31G* theoretical level calculations for temozolamide drug compounds alone and in the presence of HCM-Cellulose can be seen in Table 7. The changes of structural parameters in the drug temozolamide and HCM-Cellulose alone with when these two compounds are in the presence of each other shows that the change of structural parameters in the areas involved in the interaction is significant. Based on the results of the calculations, there is a direct relationship between the changes in the structural parameters and the values of the resonance energies resulting from the calculated electronic destabilization. So that the higher the amount of resonance energy caused by electron transfers in a bond, the greater the change in the structural parameters of that bond. NBO analysis of interactions (bonding-antibonding) at the theoretical level of B3LYP/6-31G* shows that for the Temozolomide/HCM-Cellulose complex, the highest resonance energies caused by the electron destabilization from the drug temozolamide to the HCM-Cellulose takes place as LP(1) O43 → σ* O21-H40 and LP(2) O43 → σ* O21-H40 transition with stabilization energies E(2) of 12.26 kcal/mol and 14.80 kcal/mol, respectively. Also, the highest resonance energies caused by electron transfers from the HCM-Cellulose to the drug temozolamide are σ O21-H40 → σ* O43-C55 and σ O21-H40 → π* O43-C55 transition with stabilization energies E(2) of 0.27 kcal/mol and 0.25 kcal/mol, respectively. In examining the changes in the structural parameters including bond lengths, bond angles and dihedral angles in the Temozolomide/HCM-Cellulose complex, it is observed that bond lengths and bond and dihedral angles related to the atoms involved in the reaction change, which can be one of the There are ways to justify the electronic interaction between temozolamide drug and HCM-Cellulose. Therefore, the difference in the structural parameters in the temozolamide drug-cellulose complex functionalized with reactive raw materials (HCM-Cellulose and temozolamide drug) can be explained by increasing the resonance energies caused by electronic destabilization from the drug temozolamide to the HCM-Cellulose and also by HCM-Cellulose to temozolamide drug molecule. Therefore, changes in structural parameters can be one of the ways to justify the physical absorption reaction through electronic transfers between two systems involved in the reaction.

**Table 7:** Structural parameters calculated using B3LYP/6-31G* theoretical level calculations for temozolamide drug compounds alone and in the presence of HCM-Cellulose

Optimized structure parameters / B3LYP/6-31G*				
Bond order in (ºA) within (Temozolomide)		Bond order in (ºA) within (Temozolomide in cellulose)		|Δr|
C(10)-C(14)	1.492	C(51)-C(55)	1.4809	0.011
N(8)-C(14)	1.362	N(49)-C(55)	1.3522	0.010
N(4)-N(7)	1.396	N(45)-N(46)	1.3883	0.008
O(2)-C(14)	1.222	O(43)-C(55)	1.2371	0.014

Bond angle in (º) within (Temozolomide)		Bond angle in (º) within (Temozolomide in cellulose)		|Δθ|
C(10)-C(14)-N(8)	112.974	C(51)-C(55)-N(49)	120.487	7.512
H(18)-C(13)-H(16)	110.228	H(59)-C(54)-H(57)	123.672	13.443
H(18)-C(13)-N(4)	109.485	H(59)-C(54)-N(45)	122.583	13.098
H(15)-C(12)-N(5)	127.016	H(56)-C(53)-N(48)	109.612	17.403
H(15)-C(12)-N(3)	122.081	H(56)-C(53)-N(44)	109.737	12.343
N(4)-C(11)-N(3)	110.152	N(48)-C(52)-N(45)	127.159	17.007
N(3)-C(11)-O(1)	123.424	N(45)-C(52)-O(42)	110.726	12.698
C(14)-C(10)-C(9)	128.474	C(55)-C(51)-C(50)	107.157	21.316
C(9)-C(10)-N(5)	109.483	C(50)-C(51)-N(44)	114.902	5.418
C(10)-C(9)-N(6)	134.078	C(51)-C(50)-N(48)	126.682	7.395
C(10)-C(9)-N(3)	104.981	C(51)-C(50)-N(47)	110.577	5.596
H(20)-N(8)-C(14)	118.564	H(61)-N(49)-C(55)	129.141	10.576
N(6)-N(7)-N(4)	119.967	C(53)-N(48)-C(52)	109.610	10.357
C(12)-N(5)-C(10)	107.241	C(52)-N(48)-C(50)	119.490	12.248
C(13)-N(4)-C(11)	119.944	C(50)-N(47)-N(46)	133.835	13.891
C(13)-N(4)-N(7)	113.252	N(47)-N(46)-N(45)	104.944	8.308
C(11)-N(4)-N(7)	126.803	C(54)-N(45)-C(52)	121.221	5.582

Dihedral angle in (º) within (Temozolomide)		Dihedral angle in (º) within (Temozolomide in cellulose)		|Δφ|
N(5)-C(10)-C(14)-O(2)	179.983	N(44)-C(51)-C(55)-O(43)	167.933	12.049
N(5)-C(10)-C(14)-N(8)	0.022	N(44)-C(51)-C(55)-N(49)	− 12.665	12.687
C(9)-C(10)-C(14)-O(2)	− 0.011	C(50)-C(51)-C(55)-O(43)	− 9.331	9.320
C(9)-C(10)-C(14)-N(8)	− 179.972	C(50)-C(51)-C(55)-N(49)	− 170.069	9.903
H(19)-N(8)-C(14)-O(2)	− 179.974	H(60)-N(49)-C(55)-O(43)	− 174.982	4.992
H(19)-N(8)-C(14)-C(10)	− 0.014	H(60)-N(49)-C(55)-C(51)	5.609	5.623
H(20)-N(8)-C(14)-O(2)	0.018	H(61)-N(49)-C(55)-O(43)	− 5.998	6.017
H(20)-N(8)-C(14)-C(10)	179.978	H(61)-N(49)-C(55)-C(51)	174.592	5.385
N(7)-N(4)-C(13)-H(16)	179.994	N(46)-N(45)-C(54)-H(57)	130.156	49.837
N(7)-N(4)-C(13)-H(17)	59.967	N(46)-N(45)-C(54)-H(58)	109.356	49.389
N(7)-N(4)-C(13)-H(18)	− 59.973	N(46)-N(45)-C(54)-H(59)	− 10.078	49.895
C(11)-N(4)-C(13)-H(16)	0.004	C(52)-N(45)-C(54)-H(57)	51.397	51.393
C(11)-N(4)-C(13)-H(17)	− 120.023	C(52)-N(45)-C(54)-H(58)	− 69.089	50.933
C(11)-N(4)-C(13)-H(18)	120.035	C(52)-N(45)-C(54)-H(59)	171.475	51.439
C(13)-N(4)-C(11)-O(1)	− 0.016	C(54)-N(45)-C(52)-O(42)	− 5.450	5.433
C(13)-N(4)-C(11)-N(3)	− 179.993	C(54)-N(45)-C(52)-N(48)	− 174.203	5.790
C(13)-N(4)-N(7)-N(6)	180.000	C(54)-N(45)-N(46)-N(47)	174.519	5.480
C(12)-N(3)-C(11)-N(4)	179.995	C(53)-N(48)-C(52)-N(45)	174.784	5.211

### Electronic structure and excited states

**Fig. 7:**
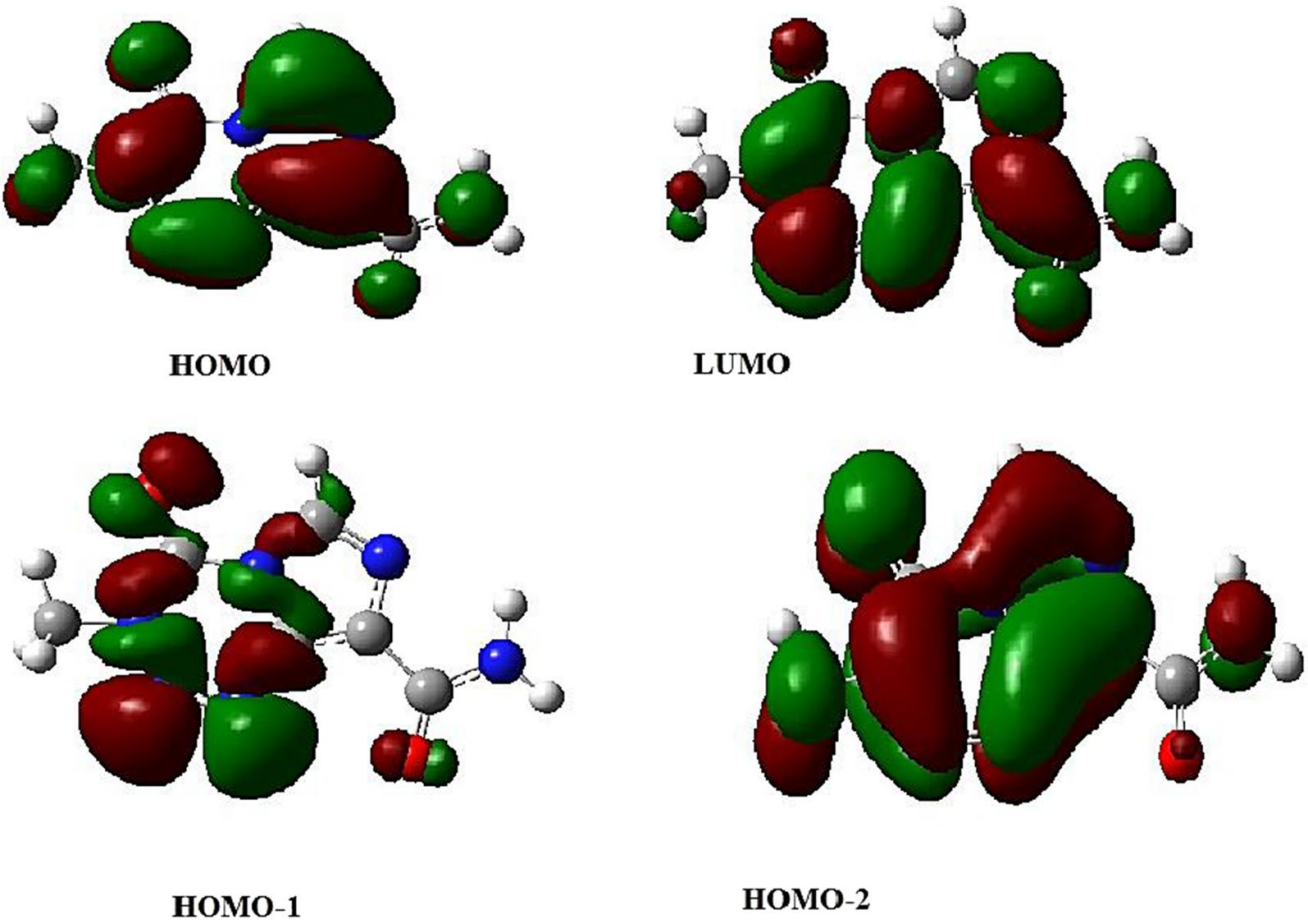
The MOs responsible for producing Temozolomide's absorption spectrum at $\mathrm{\lambda max}= 294.02nm$

**Fig. 8:**
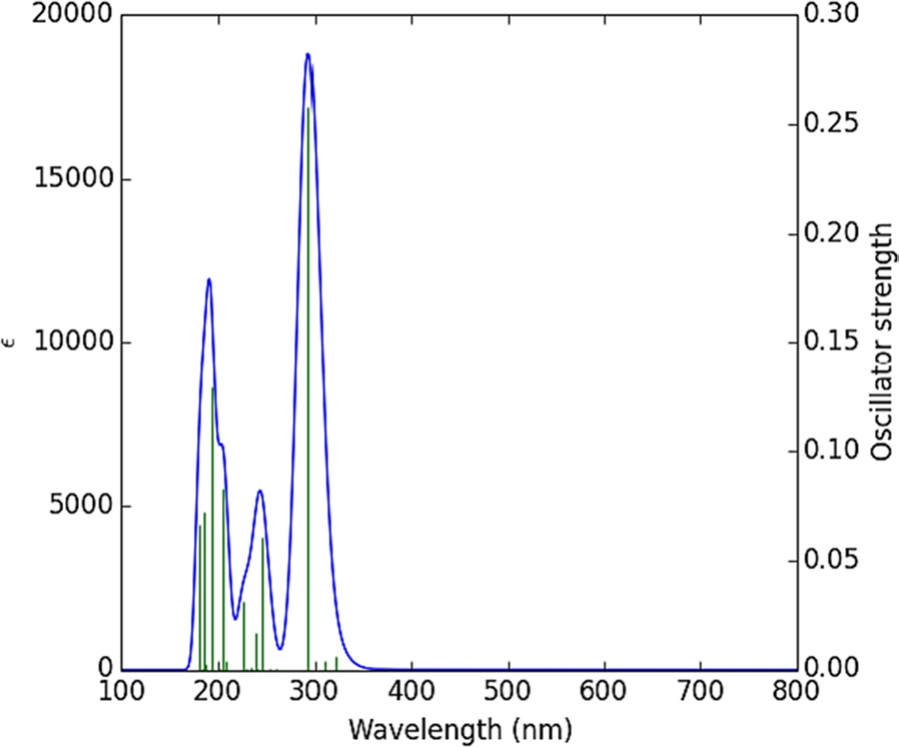
UV spectrum of the compound Temozolomide

A theoretical absorption spectrum of selected compounds in the gas phase was calculated using the TDB3LYP/6-31G* td = (nstates = 20,root = 1) method. For the calculation, 20 excited states were considered. (Table 8) shows that the Temozolomide compound has a strong absorption peak at $\lambda max= 294.02$ nm at oscillator strength f = 0.258. Maximum wavelengths formed at 294.02 nm are the result of electron charge transfer into excited state 4 and two electron excitation configurations [(H-1 > LUMO) and (H-2 > LUMO)]. The shape molecular orbitals participants are shown in Fig. 7 at $\lambda max=294.02$ nm. As shown in Fig. 7, electron density of HOMO-1 orbital spreads over the tetrazine ring and lone pairs of oxygen atoms and electron density of HOMO-2 orbital speared over lone pairs of oxygen and nitrogen atoms. The LUMO orbital electron density is located on the double bonds of Temozolomide drug. Thus, pi (*) bonds and lone pairs are responsible for this electronic transition between HOMO-1 and HOMO-2 to LUMO. A second example of an important excited state occurs at 193.47 nm (f = 0.1294) with two important electronic excitation configurations [(H-2- > L + 2) and (H-1- > L + 2)], (Table 8) shows that all other excited states of Temozolomide have very small intensities (f ≈ 0), which are nearly forbidden by orbital symmetry considerations. According to (Fig. 8), the calculated electronic absorption spectrum of Temozolomide appears in gas phase.

**Table 8:** Electronic absorption spectrum of the compound Temozolomide

Excited state	Wavelength (nm)	Excitation Energy (Cm^−1^)	Configurations Composition (corresponding transition orbitals)	Oscillator Strength (f)
S1	371.97	26,883.27	HOMO- > LUMO (97%)	0.0001
S2	321.37	31,116.06	H-3- > LUMO (98%)	0.0061
S3	310.10	32,246.85	H-2- > LUMO (64%), H-1- > LUMO (35%)	0.0036
S4	294.02	34,010.79	H-2- > LUMO (34%), H-1- > LUMO (57%)	0.258
S5	261.37	38,259.72	HOMO- > L + 1 (96%)	0.0001
S6	253.84	39,393.73	H-4- > LUMO (10%), H-1- > L + 1 (78%), H-7- > LUMO (7%), H-1- > LUMO (4%)	0.0002
S7	246.12	40,630.18	H-5- > LUMO (11%), H-1- > L + 1 (78%)	0.0604
S8	241.66	41,380.27	H-6- > LUMO (78%), H-3- > L + 1 (10%), HOMO- > L + 2 (4%)	0.0003
S9	239.64	41,728.71	H-4- > LUMO (28%), H-2- > L + 1 (65%),H-7- > LUMO (2%), H-1- > L + 1 (2%)	0.017
S10	234.80	42,589.3	H-7- > LUMO (11%), H-4- > LUMO (49%), H-2- > L + 1 (33%), H-1- > L + 2 (3%)	0.0013
S11	230.18	43,442.63	H-3- > L + 1 (81%), H-6- > LUMO (9%), H-6- > L + 1 (2%), HOMO- > L + 2 (4%)	0.0003
S12	227.23	44,008.03	HOMO- > L + 2 (85%), H-6- > LUMO (8%)	0.031
S13	209.31	47,775.45	H-7- > LUMO (67%), H-4- > LUMO (9%), H-1- > L + 1 (9%), H-1- > L + 2 (8%)	0.0039
S14	206.02	48,536.83	H-6- > L + 1 (21%), H-5- > L + 1 (71%), H-3- > L + 1 (4%)	0.0829
S15	201.61	49,600.68	H-7- > LUMO (10%), H-1- > L + 2 (79%), H-2- > L + 2 (6%)	0.0007
S16	193.47	51,685.62	H-2- > L + 2 (31%), H-1- > L + 2 (55%)	0.1294
S17	187.31	53,385.84	H-2- > L + 2 (89%), H-1- > L + 2 (5%)	0.0024
S18	186.51	53,614.9	H-4- > L + 1 (90%), H-7- > L + 1 (2%)	0.0724
S19	183.62	54,460.17	H-8- > LUMO (11%), H-3- > L + 2 (86%)	0.0005
S20	180.76	55,321.57	H-8- > LUMO (83%), H-3- > L + 2 (11%)	0.0662

**Fig. 9:**
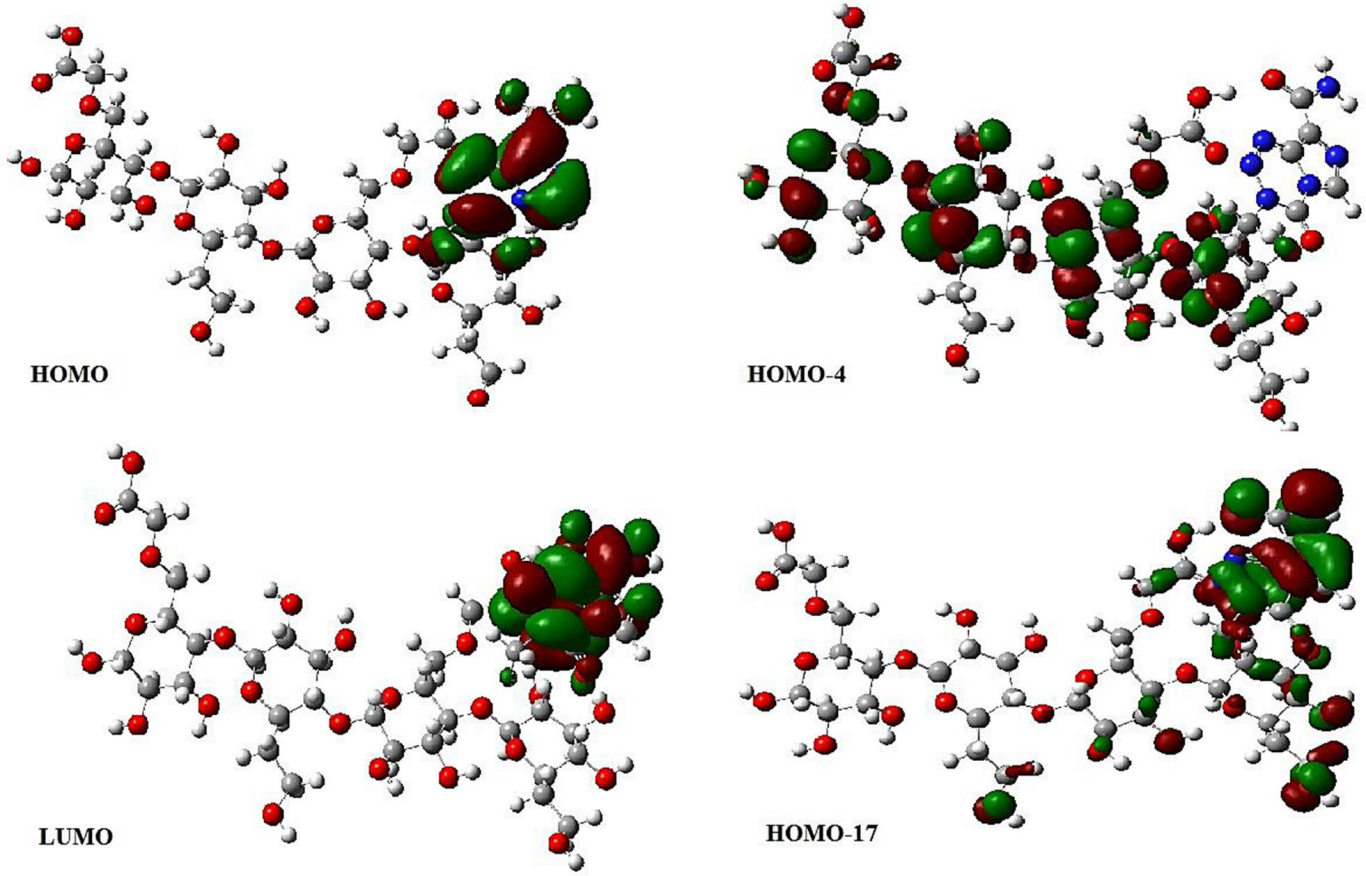
The MOs responsible for producing of the Temozolomide/HCM-Cellulose's absorption spectrum at $\lambda max= 307.64 nm$

**Fig. 10:**
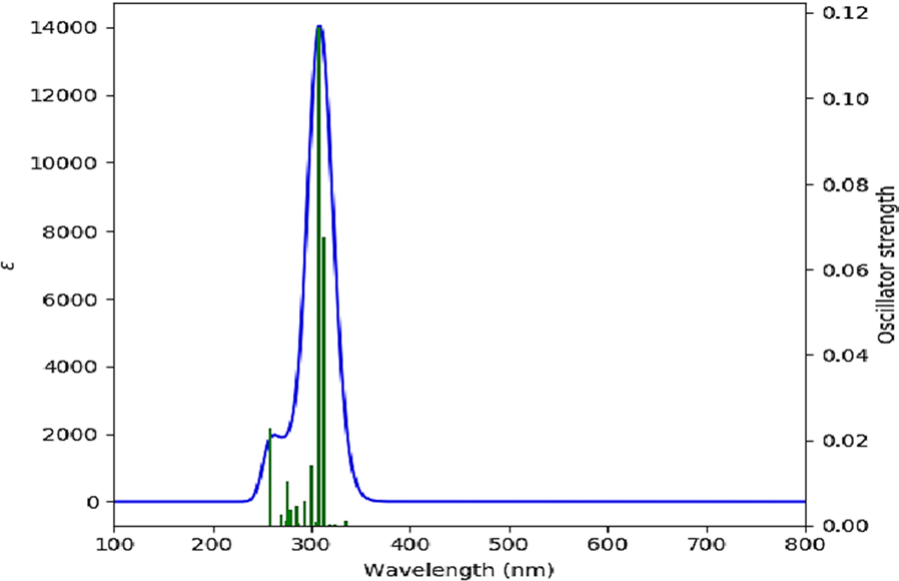
UV–vis spectrum of the complex Temozolomide/HCM-Cellulose

As summarized in (Table 9), the strongest absorption band in the Temozolomide/HCM-Cellulose complex absorption spectrum depends on the charge transfer into the S6 excited state and is defined by two configurations for one-electron excitations [(H-4- > LUMO) and (H-17 > LUMO)]. Maximum wavelength at 307.64 nm is mainly due to the transition from HOMO-4 to LUMO (Fig. 9). HOMO-4 electron densities are mainly spread over the entire HCM-Cellulose structure. The electron density of HOMO-17 is distributed across the entire Temozolomide structure and the oxygen atoms of the HCM-Cellulose substrate located near the drug. In contrast, the LUMO orbital is spread over the entire Temozolomide structure, as shown in Fig. 9. In addition to the above excited states, S4 also exhibits three configurations of electronic excitations at 313.43 nm (f = 0.0676): (H-4 > LUMO), (H-17 > LUMO), and (HOMO-21 > LUMO). Based on orbital symmetry considerations, the other excited states of the title compound are nearly forbidden by their intensity (f ≈ 0). According to (Fig. 10), the Temozolomide/HCM-Cellulose complex exhibited a calculated electronic absorption spectrum in the gas phase (UV–Vis). It is reported that the maximum wavelength of the compound Temozolomide is 294.02 nm, while the maximum wavelength of the compound Temozolomide is increased to 307.64 nm after interacting with the HCM-Cellulose. As a result, Temozolomide adsorbs onto HCM-Cellulose and changes its value $\lambda max$.

**Table 9:** Electronic absorption spectrum of the Temozolomide/HCM-Cellulose complex

Excited state	Wavelength (nm)	Excitation Energy (Cm^−1^)	Configurations Composition (corresponding transition orbitals)	Oscillator Strength (f)
S1	334.54	29,890.91	H-3- > LUMO (11%), H-1- > LUMO (73%), HOMO- > LUMO (12%), H-5- > LUMO (3%)	0.0011
S2	323.80	30,882.97	HOMO- > LUMO (86%), H-3- > LUMO (6%), H-1- > LUMO (7%)	0.0001
S3	318.90	31,357.22	H-3- > LUMO (75%), H-1- > LUMO (19%), H-5- > LUMO (4%)	0.0001
S4	313.43	31,904.87	H-21- > LUMO (11%), H-17- > LUMO (40%), H-4- > LUMO (34%), H-23- > LUMO (3%), H-18- > LUMO (5%)	0.0676
S5	311.18	32,134.74	H-2- > LUMO (99%)	0.0
S6	307.64	32,504.95	H-17- > LUMO (24%), H-4- > LUMO (53%), H-23- > LUMO (4%), H-21- > LUMO (7%), H-4- > L + 1 (2%)	0.1166
S7	304.78	32,809.83	H-7- > LUMO (15%), H-5- > LUMO (62%), H-11- > LUMO (6%), H-8- > LUMO (2%), H-4- > LUMO (5%), H-3- > LUMO (4%)	0.0009
S8	299.63	33,373.61	H-7- > LUMO (55%), H-5- > LUMO (23%), H-12- > LUMO (6%), H-11- > LUMO (2%), H-8- > LUMO (5%), H-3- > LUMO (3%)	0.0141
S9	293.66	34,051.92	H-17- > LUMO (11%), H-15- > LUMO (28%), H-11- > LUMO (10%), H-7- > LUMO (12%), H-23- > LUMO (7%), H-21- > LUMO (9%), H-18- > LUMO (2%), H-14- > LUMO (7%), H-10- > LUMO (2%), H-9- > LUMO (5%)	0.0058
S10	289.41	34,552.79	H-8- > LUMO (15%), H-6- > LUMO (80%), H-7- > LUMO (3%)	0.0
S11	287.05	34,836.70	H-8- > LUMO (66%), H-6- > LUMO (18%), H-7- > LUMO (7%)	0.0004
S12	285.71	35,000.43	H-23- > LUMO (20%), H-21- > LUMO (12%), H-17- > LUMO (10%), H-12- > LUMO (18%), H-11- > LUMO (13%), H-9- > LUMO (11%), H-10- > LUMO (6%), H-5- > LUMO (2%)	0.0046
S13	279.72	35,749.72	H-12- > LUMO (51%), H-11- > LUMO (20%), H-23- > LUMO (3%), H-21- > LUMO (2%), H-17- > LUMO (3%), H-15- > LUMO (3%), H-9- > LUMO (2%), H-8- > LUMO (8%), H-7- > LUMO (4%)	0.0037
S14	276.75	36,132.83	H-15- > LUMO (26%), H-14- > LUMO (17%), H-11- > LUMO (37%),H-23- > LUMO (4%), H-21- > LUMO (3%), H-17- > LUMO (2%), H-13- > LUMO (3%), H-12- > LUMO (4%)	0.0103
S15	275.14	36,344.15	H-12- > LUMO (11%), H-9- > LUMO (62%), H-15- > LUMO (8%), H-14- > LUMO (3%), H-13- > LUMO (4%), H-11- > LUMO (2%), H-10- > LUMO (5%)	0.001
S16	269.17	37,149.9	H-19- > LUMO (16%), H-18- > LUMO (68%), H-23- > LUMO (3%), H-17- > LUMO (5%), H-15- > LUMO (2%)	0.0025
S17	267.51	37,380.57	H-10- > LUMO (83%), H-9- > LUMO (13%), H-12- > LUMO (3%)	0.0
S18	264.04	37,871.76	H-15- > LUMO (14%), H-14- > LUMO (33%), H-13- > LUMO (44%), H-11- > LUMO (5%)	0.0
S19	263.38	37,966.94	H-15- > LUMO (12%), H-14- > LUMO (36%), H-13- > LUMO (48%)	0.0
S20	258.89	38,625.09	H-23- > LUMO (39%), H-21- > LUMO (49%), H-20- > LUMO (6%)	0.0227

## Conclusion

In this work, the nonbonding interaction of Temozolomide anticancer drug (7-Chloro-3-hydroxy-5-phenyl-1,3-dihydro-1,4-benzodiazepin-2(3H)-one) and the HCM-Cellulose nanocarrier at the B3LYP/6-31G* level of theory have been studied. The atomic charges were changed with nonbonding interaction of the Temozolomide on the HCM-Cellulose. The dipole moment of Temozolomide/HCM-Cellulose increased to 6.8985 Debye compared to Temozolomide drug alone. Based on theoretical absorption spectra, comparison between the compound Temozolomide (λmax = 294.02 nm) and the Temozolomide/HCM-Cellulose complex (λmax = 307.64 nm) has shown a bathochromic shift. According to NBO analysis of the Temozolomide/HCM-Cellulose complex, the charge distribution in Temozolomide changes when HCM-Cellulose is attached to it. Additionally, Temozolomide acts as an electron donor and HCM-Cellulose acts as an electron acceptor. Adsorption of Temozolomide on HCM-Cellulose changes the electronic properties, stability, polarity, reactivity and chemical shift tensors. This study has established the possibility of the use of HCM-Cellulose for Temozolomide delivery to the cancer diseases. Brief mentions of possible future research directions or potential improvements to the current study include:

(1) Examining the temozolamide-cellulose drug complex and comparing the adsorption of functionalized temozolamide-cellulose nanoparticles in gas and solvent phases. (2) Examining electron transfers of other anticancer drugs on HCM-Cellulose (3) Investigating the effect of organic solvents on HCM-Cellulose. (4) Examining the parameters of temperature, pressure, pH on the designed Temozolomide/HCM-Cellulose complex.

### Supplementary Information

**Additional file 1.** Full geometry optimization at the B3LYP/6-31G(d) level of approximation.

## Data Availability

The datasets used and/or analyzed during the current study are available from the corresponding author on reasonable request.
