# Supplementary material for: Theoretical study of interaction between temozolomide anticancer drug and hydroxyethyl carboxymethyl cellulose nanocarriers for targeted drug delivery by DFT quantum mechanical calculation
Source: BMC Chem. 2023 Sep 14;17(1):114. doi: 10.1186/s13065-023-01029-7 (PMC10502996; doi:10.1186/s13065-023-01029-7)
Supplement: Supplementary file 1 — Additional file 1. Full geometry optimization at the B3LYP/6-31G(d) level of approximation. [file 13065_2023_1029_MOESM1_ESM.doc]

# Supplementary data

Full geometry optimization at the B3LYP/6-31G(d) level of approximation

**Temozolamide drug**

0 1

O -2.68773500 1.69928300 0.00010000

O 2.92800100 -1.73657900 0.00007100

N -0.54958400 0.83637600 0.00002200

N -2.33292900 -0.59377400 0.00009900

N 1.52351400 1.60168600 -0.00018000

N -0.23487400 -1.55029700 -0.00003300

N -1.49004800 -1.70770400 0.00003300

N 3.95628400 0.30827400 0.00010900

C 0.28600300 -0.28591900 -0.00006700

C 1.57635100 0.22733500 -0.00017400

C -1.94934300 0.73676000 0.00016500

C 0.25266500 1.94307100 -0.00007300

C -3.75355000 -0.94442100 0.00006100

C 2.87088600 -0.51516700 -0.00032400

H -0.15146800 2.94459100 -0.00004400

H -4.32804400 -0.01974400 0.00012900

H -3.98385900 -1.53217000 0.89166500

H -3.98385700 -1.53203700 -0.89163200

H 3.84440200 1.31184600 0.00018100

H 4.87609100 -0.10599600 0.00044800

**HCM-Cellulose**

0 1

O 6.80958900 -0.69756200 0.72137100

C 8.14231300 -1.19278500 0.46355300

C 9.11847900 -0.00941400 0.54943800

C 8.67298500 1.08856400 -0.40771700

C 7.23531000 1.52111000 -0.14836900

C 6.32589400 0.29172900 -0.21031800

O 5.04514200 0.66558600 0.17082700

C 8.37676700 -2.30703200 1.48328500

O 9.57419300 2.17840200 -0.27050200

O 6.92236400 2.47745600 -1.14210400

O 1.55329000 0.10229800 -0.70336700

C 2.70067000 0.85006800 -0.31668300

C 3.93203200 -0.07639800 -0.34290400

C 3.65151600 -1.32129000 0.51403700

C 2.31763700 -1.98234400 0.16003800

C 1.20605700 -0.94080600 0.21009300

O 0.01040800 -1.49959200 -0.22974800

C 2.71992000 2.02318600 -1.29725500

O 4.63317100 -2.33516200 0.37078000

O 2.04436200 -3.02487900 1.08125300

O 3.63609000 3.02103400 -0.86944900

O -3.59165100 -1.52707900 0.23660800

C -2.30317300 -1.99477700 -0.17888800

C -1.20174400 -1.07087200 0.39583000

C -1.51279800 0.40952700 0.12374200

C -2.96903700 0.74871900 0.43506100

C -3.89621200 -0.23797400 -0.26134700

O -5.21387900 0.08376300 0.09548900

C -2.23238800 -3.45242900 0.27964500

O -0.65956300 1.22673500 0.92109800

O -3.16494300 2.09572800 0.01058300

O -8.72389800 -0.34278400 -0.74265600

C -7.56975600 0.07742900 -0.02405300

C -6.27676300 -0.28324700 -0.79415600

C -6.28086300 -1.76771700 -1.18933600

C -7.62563200 -2.18490600 -1.78810900

C -8.77960600 -1.75285900 -0.89568200

O -9.96617500 -2.09542300 -1.54352400

C -7.70863300 1.57336300 0.20777000

O -5.25549400 -1.99203700 -2.15041500

O -7.56402200 -3.59419100 -1.96341500

O -8.75679300 1.78222900 1.13536000

C 3.44836100 4.23689300 -1.53772700

C 4.52750200 5.20261000 -1.08976900

C -9.00682600 3.13767200 1.37418600

C -10.14017700 3.26756300 2.37724900

O -10.38045600 4.58157600 2.62269600

O 4.20741800 6.46949500 -1.41842100

H 8.18554600 -1.60451900 -0.55824900

H 9.09851200 0.38710400 1.57750400

H 8.72668200 0.68702600 -1.43478800

H 7.15453000 1.94572700 0.86229500

H 6.32811900 -0.13682400 -1.22655500

H 7.52882300 -3.00117800 1.42727000

H 8.37294700 -1.88206200 2.49406700

H 9.17606600 2.90419700 -0.78239800

H 6.20736900 3.05635200 -0.81530500

H 2.57864200 1.25271900 0.70092000

H 4.13673200 -0.38560200 -1.37834200

H 3.59540500 -0.98729300 1.56374000

H 2.36746300 -2.36713000 -0.87029600

H 1.10002100 -0.52720300 1.22272800

H 1.69724100 2.42648600 -1.32200000

H 2.97148800 1.67567200 -2.31177300

H 5.47990400 -1.95859700 0.69001100

H 2.88447900 -3.50910800 1.16843700

H -2.23899800 -1.94813900 -1.27814900

H -1.13133700 -1.21741000 1.48324900

H -1.33441600 0.60066300 -0.94576500

H -3.13933000 0.65074500 1.51741700

H -3.77035100 -0.20867400 -1.35316400

H -3.14813400 -3.93663800 -0.07961100

H -2.26473000 -3.49178400 1.37528400

H -0.98442900 2.13462300 0.79791400

H -4.08093500 2.32397500 0.23495400

H -7.52969400 -0.42252500 0.95635600

H -6.20859300 0.31434500 -1.71582600

H -6.09709600 -2.36411100 -0.28402000

H -7.76013900 -1.67146700 -2.75230200

H -8.70804800 -2.23177700 0.09775500

H -10.70593300 -1.84543600 -0.96634700

H -7.92989400 2.06950100 -0.75144200

H -6.76012600 1.97246000 0.59660300

H -5.32350200 -2.93074700 -2.39345100

H -8.40843800 -3.86498900 -2.35882600

H 3.53032000 4.13032500 -2.63435000

H 2.46044800 4.67900500 -1.33321800

H -8.12402200 3.66095500 1.78109700

H -9.29632000 3.67590500 0.45535000

H -11.11278600 4.60757100 3.26663400

H 4.95916000 7.02968600 -1.14606800

O -10.75353400 2.36858300 2.89389400

O 5.56152200 4.90129700 -0.53483100

O 10.42464800 -0.45185500 0.20581800

H 10.94512100 0.35673100 0.06052800

C -1.04918100 -4.27083600 -0.23115200

H -0.11944100 -3.97863100 0.26664200

H -0.91522800 -4.10265400 -1.31295800

O -1.36801200 -5.64126700 0.03238400

H -0.55212100 -6.15183300 -0.07591300

C 9.64900000 -3.12622100 1.27776100

H 9.71072700 -3.46697300 0.23097200

H 10.53757000 -2.51612400 1.47556200

O 9.56134300 -4.23417200 2.17334400

H 10.39982600 -4.71692900 2.12393300

**Temozolomide/HCM-Cellulose complex**

0 1

O 5.03280000 -2.48029300 0.32541700

C 6.24449100 -3.23524100 0.09265200

C 7.39521500 -2.24351700 -0.13364000

C 7.03797100 -1.30572500 -1.27905700

C 5.70062800 -0.60511300 -1.07467200

C 4.61000100 -1.63342800 -0.76434900

O 3.46921300 -0.95877500 -0.34800100

C 6.40272800 -4.14884000 1.30818100

O 8.09019400 -0.35144900 -1.41387400

O 5.43538700 0.11689600 -2.25811600

O -0.13379500 -0.93355700 -0.89345100

C 1.14803300 -0.37505100 -0.62838800

C 2.19079700 -1.50799900 -0.68701600

C 1.78076400 -2.61158300 0.29847300

C 0.33575600 -3.06445300 0.07994100

C -0.58359300 -1.84638400 0.11019700

O -1.88035300 -2.22513000 -0.21958900

C 1.30810400 0.74693100 -1.65509100

O 2.58235800 -3.77943700 0.20146800

O -0.03929700 -3.97540100 1.09996300

O 2.46881700 1.50226000 -1.35686500

O -5.40484200 -1.56470300 0.35061800

C -4.23700000 -2.28857800 -0.05217800

C -2.96404900 -1.54236600 0.41768200

C -3.01476000 -0.04908200 0.05696100

C -4.37561700 0.56656900 0.37589800

C -5.48455600 -0.27880600 -0.23515000

O -6.71463600 0.29839600 0.11287500

C -4.41095600 -3.69349500 0.52816900

O -2.00343200 0.64464500 0.78362900

O -4.33999600 1.89739200 -0.13385100

O -10.24389400 0.52827200 -0.72240900

C -9.02729000 0.74891100 -0.01761100

C -7.83499900 0.09714900 -0.75793500

C -8.13485000 -1.37793000 -1.06480000

C -9.53634200 -1.55140400 -1.65381400

C -10.58026300 -0.84851300 -0.79906500

O -11.81159500 -0.98262200 -1.44015500

C -8.86558800 2.25272500 0.13465600

O -7.17696900 -1.86052400 -1.99985500

O -9.75799000 -2.95214800 -1.75144400

O -9.85346900 2.71420400 1.03683800

C 2.46235700 2.79888500 -1.90201400

C 3.82959000 3.40869200 -1.62713400

C -9.82829700 4.10279700 1.20421300

C -10.91718700 4.50737900 2.18303900

O -10.89074800 5.85358600 2.36037100

O 3.77022700 4.70997600 -1.41414000

H 6.12204600 -3.84279100 -0.81915800

H 7.53851600 -1.65447100 0.78396800

H 6.96695000 -1.90758700 -2.20072100

H 5.76977400 0.06402300 -0.20681600

H 4.40656300 -2.25607900 -1.64985400

H 5.45325700 -4.67813600 1.45822400

H 6.57238200 -3.53777400 2.20255600

H 7.77920200 0.26684700 -2.09906300

H 4.96616800 0.94665000 -2.02430700

H 1.17219100 0.07913300 0.37484100

H 2.22366900 -1.91851800 -1.70613200

H 1.84999100 -2.18647700 1.31408000

H 0.24705700 -3.52598000 -0.91561300

H -0.55141500 -1.35960100 1.09499700

H 0.40378600 1.36886300 -1.58642200

H 1.35974100 0.33538700 -2.67546600

H 3.48922500 -3.52482100 0.47094200

H 0.71056600 -4.59158100 1.17482900

H -4.20605800 -2.34149700 -1.15236300

H -2.86794100 -1.63095600 1.50959500

H -2.83855500 0.04349700 -1.02558800

H -4.52542300 0.56789500 1.46564000

H -5.38332300 -0.34759200 -1.32752000

H -5.41001100 -4.03323900 0.23070800

H -4.41128000 -3.63612400 1.62352000

H -2.15128700 1.58682600 0.59537700

H -5.18783500 2.30576500 0.10199900

H -9.08496900 0.30411600 0.98831100

H -7.65656500 0.61447200 -1.71291400

H -8.07341800 -1.94330500 -0.12366000

H -9.56495600 -1.07553900 -2.64570700

H -10.60566200 -1.27621200 0.21961500

H -12.48564200 -0.55570600 -0.88672600

H -8.98091600 2.73222700 -0.85125000

H -7.85753400 2.47458700 0.51506300

H -7.43694900 -2.77701400 -2.19320800

H -10.63990900 -3.07044100 -2.13999500

H 2.30795100 2.78598700 -2.99549800

H 1.67685200 3.42834900 -1.46077700

H -8.86045600 4.45974500 1.59741300

H -10.00027300 4.63969200 0.25558200

H -11.60573100 6.05796800 2.99176000

H 4.68536700 5.06522800 -1.21366000

O -11.70052400 3.77644100 2.73397000

O 4.86053800 2.75021700 -1.62826700

O 8.57632800 -2.97060500 -0.45649200

H 9.20812300 -2.31070300 -0.78825400

C -3.40887100 -4.74932000 0.06846100

H -2.42180900 -4.58082500 0.51053700

H -3.29388400 -4.70563800 -1.02756100

O -3.94699400 -6.01297200 0.47157400

H -3.24022000 -6.66874100 0.37890000

C 7.49903800 -5.20561800 1.19524800

H 7.39987100 -5.74835200 0.24067100

H 8.48934700 -4.73669500 1.20351000

O 7.32375500 -6.08519000 2.30587800

H 8.06561800 -6.70823000 2.30501400

O 7.49154700 0.29831500 2.36238100

O 6.20113000 5.81574900 -1.01697000

N 7.45391300 2.27001900 1.15749700

N 5.58211600 1.59502900 2.29302000

N 8.76881800 3.39118200 -0.21769600

N 5.45650600 3.61028400 1.19605800

N 4.89614000 2.74771700 1.93518100

N 8.42494300 5.81189200 -1.48070600

C 6.72704900 3.38673600 0.74571200

C 7.57322500 4.06582000 -0.11713600

C 6.89598300 1.28452700 1.98388400

C 8.67555500 2.32176600 0.54072400

C 4.79247600 0.61486600 3.04335500

C 7.31726300 5.29480200 -0.90256800

H 9.41662600 1.54827800 0.67057900

H 5.33976200 0.30721200 3.93623900

H 4.59392000 -0.25855200 2.41510600

H 3.85742200 1.10357800 3.31383100

H 9.30254700 5.31299900 -1.42463100

H 8.32355300 6.60200700 -2.10099600
